# Cardiovascular aging: from cellular and molecular changes to therapeutic interventions

**DOI:** 10.20517/jca.2023.09

**Published:** 2023-05-04

**Authors:** Angeliki Vakka, Junco S. Warren, Konstantinos Drosatos

**Affiliations:** 1 Metabolic Biology Laboratory, Cardiovascular Center, Department of Pharmacology and Systems Physiology, University of Cincinnati College of Medicine, Cincinnati, OH 45267, USA.; 2 Center for Vascular and Heart Research, Department of Human Nutrition, Fralin Biomedical Research Institute at Virginia Tech Carilion, Virginia Tech, Roanoke, VA 24016, USA.

**Keywords:** Cardiovascular aging, oxidative stress, mitochondrial dysfunction, autophagy, inflammaging, fibrosis

## Abstract

Progressive age-induced deterioration in the structure and function of the cardiovascular system involves cardiac hypertrophy, diastolic dysfunction, myocardial fibrosis, arterial stiffness, and endothelial dysfunction. These changes are driven by complex processes that are interconnected, such as oxidative stress, mitochondrial dysfunction, autophagy, inflammation, fibrosis, and telomere dysfunction. In recent years, the advances in research of cardiovascular aging, including the wide use of animal models of cardiovascular aging, elucidated an abundance of cell signaling pathways involved in these processes and brought into sight possible interventions, which span from pharmacological agents, such as metformin, sodium-glucose cotransporter 2-inhibitors, rapamycin, dasatinib and quercetin, to lifestyle changes.

## INTRODUCTION

Cardiovascular disease (CVD) is the major cause of death, and, specifically in 2020, it led to approximately 19.1 million deaths globally^[1]^. According to the American Heart Association, the prevalence of CVD (including coronary heart disease, heart failure, stroke, and hypertension) in men and women in the US is ~38% for people 40–59 years old, ~73% for 60–79 years old, and ~80%−85% for 80 years and above^[2]^, indicating a strong association between aging and incidence of CVD. These statistics, in light of the projection that the number of people aged 65 years or older will rise from 10% in 2022 to 16% in 2050^[3]^, signify the importance of deciphering the mechanisms of cardiovascular aging, which will identify potential targets for therapeutic interventions.

**Figure 1.**
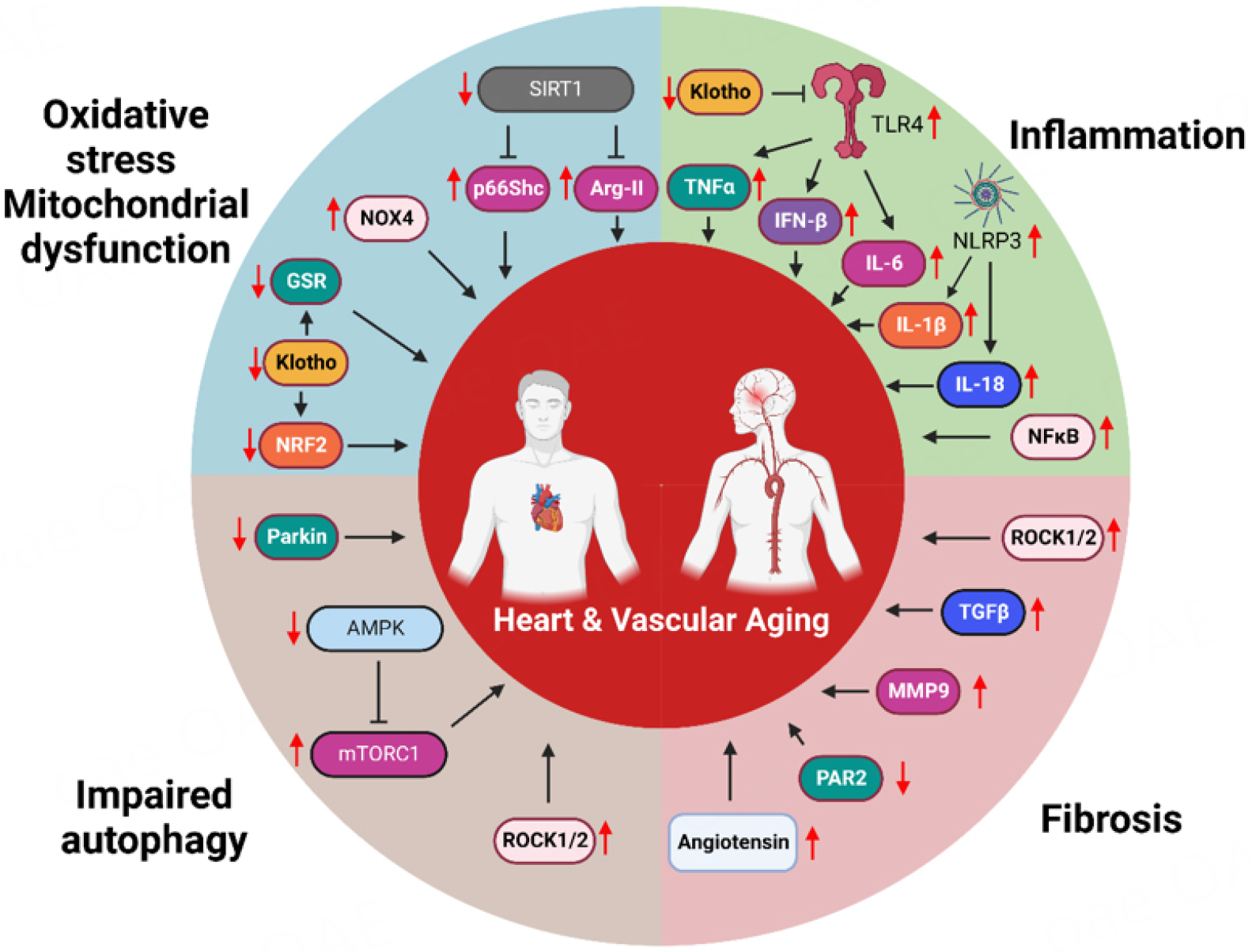
Cellular processes and signaling pathways that are common in myocardial and vascular aging. Oxidative stress and mitochondrial dysfunction in the aged cardiovascular system are associated with decreased SIRT1 levels, which increases p66Shc and Arg2 levels, as well as the expression of NOX4, while it decreases Klotho levels, resulting in higher NRF2 proteolysis. Aging decreases AMPK activation in the cardiovascular system, leading to increased mTORC1 activity, increases ROCK1 and ROCK2, and decreases Parkin, resulting in impaired autophagy. Assembly of NLRP3 inflammasome, and elevated expression of TLR4 and NF-κΒ in the aged heart and arteries result in higher production of inflammatory cytokines, while the increased levels of Ang II, ROCK1 and ROCK2, and the decreased levels of PAR2 are associated with age-related cardiac and arterial fibrosis; image created on biorender.com.

Aging leads to progressive structural and functional deterioration of the cardiovascular system. Specifically, age-related changes in the heart and vasculature include cardiac hypertrophy, diastolic dysfunction, myocardial fibrosis, arterial stiffness, and endothelial dysfunction^[4–8]^. Major cellular hallmarks of cardiovascular aging include oxidative stress, mitochondrial dysfunction, reduced autophagy, inflammation, and telomere dysfunction^[9–11]^. Some of the molecular mechanisms that underlie these processes are involved in causing both myocardial and vascular aging [Figure 1]. To identify these molecular mechanisms and discover potential therapeutic targets, animal models of natural and experimental-induced aging are widely used.

In this article, we review the structural and functional changes in the aged cardiovascular system and the underlying signaling pathways that are involved in cardiovascular aging, and discuss interventions that have been tested at the preclinical and clinical levels.

## CLINICAL MANIFESTATIONS OF AGING IN THE CARDIOVASCULAR SYSTEM

### Aging vasculature

Age-associated vascular changes include arterial stiffening and endothelial dysfunction. Large elastic arteries, such as the central aorta and carotid artery, exhibit age-related dilatation, leading to an increase in lumen diameter, and wall thickening mainly in the intima^[12]^. Arterial wall thickness is associated with arterial stiffening, which is caused by the loss of central arteries’ elastin lamellae and its replacement by collagen, leading to elevation of systolic pressure and lowering of diastolic pressure^[13]^. In addition to structural changes, arterial stiffness is also associated with age-related endothelial dysfunction, because aged endothelium is characterized by decreased antithrombotic and vasodilatory capacity due to chronic inflammation and oxidative stress^[14]^.

### Ventricular and atrial changes

The vasculature-driven changes in systolic pressure lead to left ventricular (LV) afterload rise, LV hypertrophy, and increased myocardial requirements for oxygen^[15]^. Concomitantly, decreased diastolic pressure compromises myocardial perfusion and may result in myocardial ischemia, since coronary perfusion occurs during diastole rather than systole^[16]^. The Framingham Heart Study and the Baltimore Longitudinal Study on Aging showed that an increase in LV wall thickness is age-dependent in healthy adults of both sexes, even without hypertension^[17,18]^. The increasing amount of cardiomyocyte death with aging forces the remaining cardiomyocytes to become hypertrophic and stimulates fibroblast proliferation, resulting in increased LV wall thickness, higher mass/volume ratio, and decreased LV end-diastolic volume^[19,20]^. These changes make the LV stiffer and less compliant. This results in a greater amount of blood during late diastolic filling via atrial contraction instead of receiving it during early diastolic filling, which is described as diastolic dysfunction^[21]^. At the same time, atrial contraction leads to atrial hypertrophy and dilation, which are associated with atrial fibrillation^[22]^. Although aging does not usually cause a decline in ejection fraction (EF), left ventricular systolic capacity is reduced under high-demanding situations such as exercise^[23]^.

### Valvular changes

The incidence of heart valve disease increases significantly with age. Specifically, an increase of moderate or severe valvular heart disease from 0.7% in people aged 18–44 years old to 13.3% in people that are 75 years or older in the US has been reported^[23]^. The most common valvular complication is mitral regurgitation, followed by aortic stenosis, aortic regurgitation, and, lastly, mitral stenosis^[23]^. Aged heart valves become stiffer due to greater matrix collagen content, collagen cross-linking, fibrosis, and lower glycosaminoglycans content^[24,25]^. Also, calcification can develop in aging heart valves, resulting in dysfunctional movements and blood flow complications^[26,27]^.

Therefore, LV afterload rise, LV hypertrophy, diastolic dysfunction, atrial fibrillation, valvular calcification, arterial stiffness, and endothelial dysfunction occur frequently in the elderly.

## CELLULAR AND MOLECULAR PROCESSES IN CARDIOVASCULAR AGING

### Oxidative stress

#### Pro-oxidant pathways in aging

The heart has a higher oxygen uptake rate and produces more reactive oxygen species (ROS) compared with other tissues in the human body^[28]^. The free radical theory of aging, first described by Denham Harman in 1956, proposes that aging and age-related diseases are caused by the harmful side attacks of free radicals on cellular components^[29]^. Further studies have shown that excessive levels of ROS, including the superoxide radical (O_2_^•−^), the hydroxyl radical (•OH), and hydrogen peroxide (H_2_O_2_), are associated with a wide range of cardiovascular diseases, such as arterial hypertension, atherosclerosis, heart failure, and atrial fibrillation^[30–33]^. Although ROS are generated by various cellular compartments, mitochondria constitute their main source during oxidative phosphorylation^[34,35]^. With age, mitochondria become dysfunctional due to the accumulation of ROS-induced damage, such as mtDNA (mitochondrial DNA) mutations, leading to elevated ROS^[36,37]^.

Evidence for the role of mitochondrial ROS in cardiac aging is provided by experiments with mice overexpressing an antioxidant, catalase targeted to mitochondria (mCAT)^[38,39]^. Constitutive mCAT expression attenuates murine cardiac aging likely due to lower mitochondrial proteome oxidation, and leads to fewer mtDNA mutations and lack of activation of the calcineurin-NFAT pathway, along with preservation of the sarcoplasmic reticulum Ca^2+^ ATPase (SERCA) pump content^[38]^. Also, late-life constitutive mCAT expression by administration of an adeno-associated virus serotype-9 vector expressing mCAT (AAV9-mCAT) improves diastolic function in aged mice^[40]^.

Mitochondrial expression of cardiac NADPH oxidase 4 (NOX4), which catalyzes the first step of ROS formation, increases with age and cardiac stress, thus becoming a main source of mitochondrial oxidative stress^[41]^. Moreover, NOX4 expression is elevated in the vasculature of old mice^[42]^. The association of NOX4 with vascular aging was established with experiments using young transgenic mice with high mitochondrial NOX4 expression, which demonstrate aortic stiffening and impaired aortic contractility similar to those observed in aged mice. Aortic vascular smooth muscle cells (VSMCs) of these mice show elevated mitochondrial H_2_O_2_ and superoxide production, increased DNA damage and suppressed expression of superoxide dismutase 2 (SOD2), the main scavenger of mitochondrial superoxide^[43]^. Furthermore, along with NOX4, phosphorylated and oxidative Ca^2+^/calmodulin-dependent protein kinase II (CaMKII) is increased in the hearts of old rats. NOX4/ROS-activated CaMKII phosphorylates Ryanodine receptor 2 (RYR2), a major Ca^2+^ channel protein in cardiac muscle, leading to acceleration of Ca^2+^ release and prolongation of diastolic relaxation^[44]^. Thus, this study implies a potential causality of the NOX4-CaMKII-RYR2 pathway with cardiac remodeling, mitochondrial oxidative stress, and ventricular tachyarrhythmia in aged mice.

The p66Shc protein, which is a member of the Src homologous-collagen homolog (ShcA) adaptor protein family that mediates ROS-induced mitochondrial ROS release (RIRR), has also been related to cardiovascular aging. Upon oxidative stress-induced phosphorylation^[45]^, p66Shc translocates to mitochondria, where it transfers electrons from cytochrome C to molecular oxygen, forming H_2_O_2_ and leading to apoptosis^[46]^. Mitochondria from the hearts of aged rats have increased levels of p66Shc^[47]^, while *p66shc*^−/−^ mice have higher nitric oxide bioavailability, decreased superoxide levels and are protected from age-dependent endothelial dysfunction^[48]^.

Silent information regulator 1 (SIRT1), a nicotinamide adenine dinucleotide (NAD+)-dependent class III histone deacetylase (HDAC), represses transcription of p66Shc via epigenetic changes and is downregulated during aging^[49,50]^. Activation of SIRT1 with SRT1720, a specific SIRT1 activator, alleviates vascular endothelial dysfunction in old mice by reducing oxidative stress, nuclear factor-kappa B (NF-κB) activation, and tumor necrosis factor alpha (TNF-α) levels and increasing cyclooxygenase-2 signaling^[51]^. SIRT1 activation reverses endothelial dysfunction in microvessels isolated from subcutaneous adipose tissue of old patients, which is associated with lower p66Shc and Arginase II (Arg2) levels, and increased expression of mitochondrial enzymes with antioxidant properties, such as SIRT3 and SOD2. Also, it upregulates the expression of genes involved in the mitochondrial respiratory chain, such as ATP synthase 6 (ATP6), cytochrome b (Cytb), NADH dehydrogenase 2 (ND2) and NADH dehydrogenase 5 (ND5), thus preventing mitochondrial ROS generation^[49]^.

The association of Arg2 with aging is also demonstrated with increased vascular and cardiac levels. Arg2 catalyzes hydrolysis of mitochondrial arginine to ornithine and urea and has also been associated with atherosclerosis^[52,53]^. Elevation of Arg2 levels in human senescent VSMCs has been associated with activation of extracellular signal-regulated kinase (ERK), c-Jun N-terminal kinase (JNK), and ribosomal S6 kinase 1 (S6K1) by p66Shc^[52]^, while genetic deficiency of Arg2 in mice extends lifespan mainly in females, which is associated with inhibition of p16 inhibitor of CDK4 (p16Ink4a), p66Shc, and S6K1 pathways^[54]^.

#### Antioxidant mechanisms in aging

Besides increasing pro-oxidant proteins, aging lowers the expression of the antioxidant nuclear factor erythroid 2-related factor 2 (NRF2), as it has been shown in aortas of aged rats and in cultured VSMCs of aged rhesus macaques, which contributes to vascular oxidative stress^[55]^. Besides the vasculature, NRF2 seems to be also important for preventing aging of cardiac muscle, as shown by the aggravation of D-galactose-induced oxidative stress and acceleration of cardiac aging in *Nrf2*^−/−^ mice. Moreover, protection from cardiac aging has been reported in mice treated with an NRF2 activator, CDDO-imidazolide^[56]^.

The protective role of NRF2 for cardiac aging has also been shown via the beneficial effects of a regulator of NRF2, secreted Klotho. Klotho is named after one of the three greek mythology “Fates” that controlled the thread of life of every person. Klotho incurs its anti-aging effects by upregulating NRF2 and Glutathione Reductase (GR) and preventing cardiac hypertrophy^[57]^. Aging decreases circulating Klotho levels in both mice and humans, and increases Kelch-like ECH-associated protein 1 (KEAP1) levels in isolated cardiomyocytes, which promotes NRF2 proteolysis^[57,58]^. Accordingly, treatment of aged mice with secreted Klotho reduces cardiac oxidative stress and rescues cardiac aging^[57]^.

Thus, aging alters cardiac and vascular expression of proteins that regulate redox balance, such as NOX4, CaMKII, p66Shc, Arg-II, SIRT1, Nrf2, Klotho, SOD2, SERCA, Cisd2, resulting in elevated ROS levels and mitochondrial dysfunction in the aged heart and vessels.

### Impaired autophagy

Autophagy is a cellular degradation process through which the cells recycle their own components. Autophagy decreases with age in numerous tissues, including the heart, and leads to accumulation of dysfunctional organelles, misfolded proteins, and lipofuscin granules^[59,60]^.

One of the main regulators of autophagy is the mammalian target of rapamycin (mTOR), a serine/threonine kinase that also regulates cell proliferation, protein synthesis, lifespan, and aging^[61]^. mTOR is a component of the protein complexes mTORC1 and mTORC2^[62]^. mTORC1 is a suppressor of autophagy and is sensitive to rapamycin, while mTORC2 is not sensitive to short-term rapamycin treatment. Cardiac and vascular mTOR activity is significantly increased in aged mice^[63,64]^. Accordingly, in mice that do not express glycogen synthase kinase-3α (GSK3A), which inhibits mTORC1 activity, autophagy is decreased, and cardiac aging appears early and is accompanied by cardiac hypertrophy, systolic dysfunction, and impaired diastolic relaxation^[65]^. Conversely, chronic cardiomyocyte-specific activation of an activator of mTOR signaling, protein kinase B (Akt), leads to aging-induced cardiac hypertrophy and myocardial contractile dysfunction^[63]^.

In a similar manner, suppression of the myosin light chain (MLC)/focal adhesion kinase (FAK)/Akt/mTOR signaling axis via deletion of Rho-associated coiled-coil-containing protein kinase (ROCK)1 and ROCK2 promotes autophagy and reduces cardiac fibrosis in aged mice^[66]^. ROCK 1 and ROCK2, which have a central role in regulating actomyosin cytoskeleton contractility, promote age-related aortic stiffening as well^[67]^. Interestingly, although mTORC1 seems to promote cardiac aging^[68]^, it was recently found that activation of mTORC2 alone increases autophagy and improves age-related cardiomyopathy in Drosophila^[69]^, indicating additional layers of regulation of mTOR signaling in regards to its involvement in cardiac aging.

AMPK, which is a serine/threonine protein kinase that acts as a cellular energy sensor of low intracellular ATP levels, a stimulator of autophagy in cardiomyocytes^[70]^, and an mTOR inhibitor^[71]^, also has low expression in the aged heart and arteries, which corroborates with increased mTOR signaling and reduced autophagy. AMPK deficiency exacerbates age-related cardiomyopathy^[72,73]^. Aging-induced deactivation of AMPK has been correlated with decreased SIRT1 activity. Consequently, SIRT1 activation in aged hearts activates AMPK and improves their tolerance to ischemic stress^[74]^. Moreover, combined deletion of both Akt2 (the main cardiac isoform of Akt) and AMPK suppresses autophagy and promotes aging-induced cardiac hypertrophy, interstitial fibrosis, and contractile dysfunction^[75]^.

A certain type of autophagy that targets mitochondria, thus referred to as mitophagy, is induced by PTEN-induced putative kinase protein 1 (PINK1), which accumulates on the damaged mitochondria and phosphorylates the E3 ubiquitin ligase Parkin. Parkin marks proteins on the outer mitochondrial membrane with phosphoubiquitin, leading to lysosome-mediated mitochondrial degradation^[76]^. Parkin levels are significantly decreased in the hearts of aged mice^[77]^. Inhibition of Parkin mitochondrial translocation -via p53- in the hearts of aged mice impairs mitophagy and causes cardiac dysfunction^[78]^. Conversely, overexpression of Parkin improves mitophagy and cardiac function in aged hearts^[77]^.

Thus, aging-induced alterations in the expression of proteins involved in cell proliferation, cytoskeleton contractility and protein degradation, such as mTORC1, ROCK1, ROCK2, and AMPK, lead to impaired autophagy in aged hearts and vessels.

### Aging-related alterations in metabolic homeostasis

Aging is associated with major metabolic events, including insulin resistance^[79,80]^. Although it is known that insulin-like growth factor-I (IGF-I) facilitates glucose metabolism and inhibits apoptosis of cardiomyocytes^[81,82]^, a study showed that the role of IGF-I receptor (IGF-IR) in cardiac health is age-dependent. Specifically, mice with cardiomyocyte-specific overexpression of IGF-IR show superior cardiac contractility at a young age, but pathological cardiac hypertrophy, increased LV fibrosis and reduced EF at 20 months of age. This pathological phenotype is attributed to reduced autophagy and lower oxidative phosphorylation in the heart^[83]^. Even in mice of 11 month-old, cardiomyocyte-specific inactivation of IGF-IR suppresses diastolic cardiac function to a greater extent than in their age-matched controls^[84]^. Moreover, mice with cardiomyocyte-specific expression of a dominant-negative mutant of phosphoinositide 3-kinase (PI3K) (dnPI3K), which is a downstream effector of insulin/IGF-1 signaling, have preserved cardiac function and less fibrosis at 20–24 months of age, decreased levels of oxidative stress and proinflammatory factors, and enhanced autophagy^[85]^. Thus, inhibition of IGF-IR in late life may prevent age-related cardiac changes.

Aging-related mitochondrial dysfunction is also associated with changes in intracellular calcium handling^[86]^. SERCA activity decreases due to increased oxidation, although its expression is not altered^[87,88]^. Lower SERCA activity increases calcium stagnation in cytoplasm, which prolongs diastolic relaxation^[89]^. Furthermore, CDGSH iron-sulfur domain-containing protein 2 (Cisd2), which is also important for intracellular Ca^2+^ homeostasis, decreases with aging in the heart^[90]^, leading to mitochondrial Ca^2+^ overload in cardiomyocytes that accounts for cardiac structural defects and functional decline^[91]^.

The occurrence of oxidative stress and mitochondrial damage in cardiovascular aging affects cellular and systemic metabolic homeostasis in the elderly. The advances in metabolomic methods have availed a powerful tool that allows for screening the numerous metabolites in biofluids and tissue. Leveraging mass spectrometry (MS)-based metabolomics, several studies identified altered metabolites and circulating prognostic biomarkers in the progression of various human diseases, including cancer, heart failure, and Alzheimer’s disease^[92–94]^. Animal studies have suggested that aging per se leads to alterations in plasma metabolic profile^[95,96]^. Although MS-based metabolomics study in aging is still limited, the altered plasma levels of amino acids and lipid species have been consistently reported as metabolic signatures of aging in humans and mice^[95,97,98]^. A recent study on metabolomic analysis of human serum samples from healthy individuals aged 20–70 years old^[98]^ showed that plasma levels of branched-chain amino acids (BCAA, isoleucine; leucine; valine), aspartate, 3-hydroxyisobutyrate are significantly decreased after the age of sixty. On the contrary, the plasma levels of hippuric acid are increased after this age^[98]^, which may be correlated to the enrichment of gut microbiota after the age of sixty, since hippuric acid is a metabolite deriving from the degradation of dietary polyphenols by a range of gut microbe^[99]^. However, the same study^[98]^ showed that despite the elevated levels of hippuric acid in plasma, variables related to cardiac autonomic modulation and cardiorespiratory fitness decrease with age and present the lowest values in the oldest age group (60–70 years old).

Another recent targeted metabolomics study of microbe-derived metabolites demonstrated that the plasma levels of hippuric acid are negatively correlated with peripheral artery disease^[100]^, suggesting the use of plasma hippuric acid as a biomarker that will identify a lower risk of CVD in the elderly.

According to plasma metabolomic analysis of young (3–4 months) and old mice (22 months), the circulating metabolic footprint of aging was found in the altered levels of amino acids (i.e., phenylalanine, tryptophan, taurine, isoleucine), phospholipids and organic acids (i.e., gluconic acid, citric acid)^[95]^. It remains elusive what organ(s) are responsible for releasing those metabolites into circulation in aging, as well as whether they play a role in contributing to myocardial remodeling during aging.

In summary, although various metabolic processes are compromised during aging, only recent MS-based metabolomic analyses have identified metabolites that may serve as biomarkers or potential targets of therapeutic interventions. The challenge going forward will be to design studies aiming to discover new biomarkers that can predict risks of pathogenic aging and help elucidate the critical metabolic pathways that account for the onset and progression of aging-related cardiovascular diseases.

### Inflammation

Dysregulation of the immune system in the elderly leads to chronic sterile low-grade inflammation^[101]^. This process, called “inflammaging”, is a key factor in the development of frailty and age-related degenerative diseases, including cardiovascular complications^[102,103]^. A major pathway of cardiovascular inflammaging is the NOD-like receptor protein (NLRP) 3/caspase-1 cascade^[104]^. NLRP3 is an intracellular detector of endogenous danger signals, microbial motifs and environmental irritants, leading to the formation and activation of the inflammasome. During aging, damaged mitochondria and defective autophagy lead to ROS accumulation and the release of damage-associated molecular patterns (DAMPs) that trigger the NLRP3 inflammasome^[105]^. Assembly of the NLRP3 inflammasome results in the secretion of caspase-1-dependent proinflammatory cytokines, such as IL-1β and IL-18^[106]^. In a D-galactose-induced model of cardiomyocyte aging, NLRP3 inflammasome is activated and IL-1β levels are increased, whereas inhibition of NLRP3 inflammasomes by MCC950 or N-acetylcysteine attenuates cardiomyocyte aging^[107]^. NLRP3 inflammasome also participates in vascular endothelial cell senescence via a mechanism that involves binding of TXNIP to NLRP3, which is triggered by oxidative stress^[108]^.

Moreover, increased cardiac and aortic expression of Toll-like receptor 4 (TLR4), which is a pattern recognition receptor of the innate immune system, is observed with aging^[109]^ and accounts for higher production of inflammatory cytokines, such as IFN-β, IL-1β, IL-6, and TNF-α. To this end, aged *TLR4*^−/−^ mice have lower levels of these cytokines and improved cardiac function and vascular relaxation^[109]^.

Transcriptional factor NF-κB, which is involved in TLR4 signaling and is tightly associated with inflammation, also plays an important role in cardiovascular aging^[110]^. Treatment of endothelial cells with TNF-α activates NF-κB and increases proatherogenic inflammatory mediators, such as iNOS and adhesion molecules, and incurs age-related alterations of the arterial endothelium, such as impaired acetylcholine-induced relaxation^[111,112]^. Aged hearts also have increased NF-κB signaling that mediates inflammatory response, while NF-κB inhibition alleviates cardiac inflammation, apoptosis, and age-associated LV remodeling^[113,114]^. Specifically, supplementation with the anti-aging serum soluble Klotho inhibits the TLR4/Myd88/NF-κB pathway, resulting in improved cardiac function in aged mice^[110]^.

Matrix metalloproteinase- (MMP-)9, monocyte chemotactic protein (MCP)-1 levels, and macrophage density increase in the left ventricles of senescent mice^[115]^. MMP-9 stimulates cardiac macrophage activation and proinflammatory response in aged mice, while MMP-9 deletion results in decreased proinflammatory gene expression and collagen deposition, and attenuates age-related LV diastolic dysfunction^[116]^.

Thus, NLRP3 inflammasome activation, increased TLR4 expression, elevated production of inflammatory cytokines, and increased NF-κB signaling are important processes and factors in inflammaging, which is a major cellular process involved in cardiovascular aging.

### Fibrosis

The development of myocardial fibrosis is another hallmark of cardiovascular aging. Fibroblast activation and proliferation, along with hypertrophy of myocytes, compensate for myocyte loss due to apoptosis and necrosis^[117–120]^. Fibrosis is characterized by excessive deposition of extracellular matrix by cardiac myofibroblasts, which constitute the main cardiac cell type that undergoes senescence^[121]^. Senescence in cardiac fibroblasts depends on p53/p21 and p16/Rb pathways. However, genetic ablation of p53 and p16(INK4a) (*Trp53*^−/−^ *Cdkn2a*^−/−^ mice) eliminates senescence, but it exacerbates fibrosis with pressure overload, resulting in severe cardiac dysfunction^[121]^. Furthermore, cardiac-specific induction of senescence in the same study lowered perivascular fibrosis, which contradicts findings that correlate fibrosis with cardiac aging. This finding indicates potential protective effects of isolated cellular senescence against fibrosis in a young heart as opposed to an aged heart that cellular senescence is extensive.

TGF-β signaling, which is one of the primary regulators of tissue fibrosis, is also activated in cardiac aging. Mice with heterozygous Tgfb1 deletion have decreased age-related myocardial fibrosis and improved LV compliance^[122]^. Moreover, chronic inhibition of TGF-β receptors prevents ventricular fibrotic remodeling with aging^[123]^. Surprisingly, the expression of TGF-β receptor I (TβRI) is decreased in aged murine cardiac mesenchymal stem cells (MSCs) and MSC-derived mesenchymal fibroblasts^[124]^. It remains to be elucidated whether age-related cardiac fibrosis is caused by the elevated activity of TGF-β or defects in the TGF-β signaling^[125]^.

Fibrosis is also modulated by the renin-angiotensin-aldosterone system (RAAS), which is a critical regulator of blood pressure, fluid balance, and systemic vascular resistance. Sustained RAAS activation, characterized by increased levels of angiotensin-converting enzyme (ACE), angiotensin II (Ang II) and Ang II type 1 receptor (AT1R), leads to NADPH oxidase activation, which activates MMP-2 and increases the expression of pro-fibrotic connective tissue growth factor (CTGF) and TGF-β1. This cascade of events leads to cardiac fibrosis and remodeling in aged rats^[126]^. In the thoracic aorta of old mice, the prorenin receptor (PRR) - ACE-Ang II-AT1R axis is activated, whereas the ACE2-Mas receptor (MasR) axis is inhibited. This altered expression of RAS components correlates with fibrosis and oxidative stress in the aging aorta^[127]^. Additionally, similarly to old rats, young adult animals develop cardiomyocyte hypertrophy and vascular remodeling when they are chronically treated with angiotensin II^[126]^. In contrast, mice with smooth muscle cell mineralocorticoid receptor (MR) deletion are protected from age-related vascular and cardiac stiffening and fibrosis^[128]^.

Moreover, protease-activated receptor (PAR) 2 is a significant regulator of pro-fibrotic PAR1 and TGF-β signaling in the heart^[129]^. Levels of PAR2 decrease with age in the aortas of rats with metabolic syndrome^[130]^. Aged PAR2-knockout mice exhibit cardiac fibrosis and diastolic dysfunction with higher transcription of TGF-βR and PAR1 compared to WT mice^[129]^. Treatment with vorapaxar, a PAR1 antagonist, reduces cardiac collagen deposition by 44%, inflammation, and TGF-β expression in a metabolic disease model of apolipoprotein E-knockout mice, which also demonstrate cardiac fibrosis and Heart Failure with Preserved Ejection Fraction (HFpEF)^[129]^.

Therefore, PAR2, TGF-β, and RAAS play an important role in aging-related fibrosis of the heart and vessels.

### Telomere dysfunction

Telomeres are DNA-protein complexes that cap the end of each chromosome arm in order to maintain chromosomal stability and integrity^[131]^. In proliferating tissues, telomeres become shortened with cell cycle divisions. When they reach a critical length, they cannot bind enough proteins on the cap and are sensed as exposed DNA ends, leading to proliferation arrest and senescence-associated secretory phenotype (SASP)^[132,133]^. Impairments in telomere length maintenance are related to cardiovascular function impairments. Leukocyte telomere shortening is a risk factor for CVD^[134]^. Telomerase deficient (*Terc*^−/−^) mice of generation 3 (G3) develop severe ventricular dysfunction, impaired mitochondrial biogenesis and function^[135,136]^, enhanced apoptosis, cardiomyocyte hypertrophy^[137]^, and vascular endothelial dysfunction similar to aged WT mice^[138]^. Aged mice with a deficiency in telomeric repressor activator protein 1 (Rap1) (*Rap1*^−/−^) show telomere shortening, DNA damage, lower cardiac fatty acid metabolism, and development of aging-associated cardiac structural and functional changes^[139]^.

Nevertheless, it should be noted that there are differences between the length and role of telomeres in senescence between rodents and humans. Specifically, although rodents have longer telomeres and ubiquitous telomerase expression, they have shorter lifespans than humans^[133]^. Also, in contrast with humans, telomere length does not determine the proliferative capacity of mouse cells^[140,141]^.

However, cardiomyocytes are postmitotic cells, and their ability to proliferate is limited. Thus, telomere dysfunction in cardiomyocytes is less likely due to telomere shortening^[142]^ but due to DNA damage within telomeres^[133]^, which can be caused by oxidative stress^[143]^. Specifically, length-independent telomere damage occurs in aged cardiomyocytes via the activation of the classical senescence-inducing pathways, cyclin-dependent kinase inhibitor 1 (p21CIP) and p16Ink4a, and leads to a non-canonical SASP, which induces myofibroblast activation and cardiomyocyte hypertrophy^[143]^.

Therefore, telomere dysfunction is an additional cause, beyond oxidative stress, mitochondrial dysfunction, impaired autophagy, inflammation, and fibrosis, that leads to cardiovascular aging.

## ANIMAL MODELS OF CARDIOVASCULAR AGING

To investigate the pathophysiology of cardiovascular aging and identify potential therapeutic targets, various animal models are used [Table 1]. Mice at 24 months of age, which are equivalent to 70-year-old humans, have increased left ventricular mass, enhanced fibrosis, aortic stiffness, and decreased cardiac diastolic function^[144,145]^. Rhesus monkeys (Macaca mulatta), which have a median lifespan of 25 years, are also used in gerontology research of the cardiovascular system due to their 92.5%−95% genetic homology to humans and their ability to develop cardiac fibrosis and hypertrophy^[146]^. Although the naturally aging models are most suitable for these studies, models of premature aging, artificially induced aging models, and genetically modified models are also used.

The senescence-accelerated mouse prone (SAMP) 8 is a model of accelerated aging that was developed by selective inbreeding of AKR/J mice with inherited senescence^[147]^. SAMP8 mice are especially used in studies of cardiovascular aging, since they show accelerated aging, increased cardiac fibrosis, and diastolic dysfunction at 6 months of age^[148]^. Wild mouse strain Mus musculus castaneus (CAST), which have short telomeres from birth, are also used as a model of cardiac aging, as they develop cardiac diastolic dysfunction by their 1st year of age^[149]^.

Another model of cardiovascular aging is the D-galactose-induced aging model, which uses 2–3-month-old rodents treated with D-galactose injections at doses of 100–500 mg/kg/day for 4–12 weeks^[150]^. D-galactose administration increases the levels of senescence-associated β-galactosidase (SA-β-gal) in the cardiac tissue^[151,152]^. D-galactose treatment increases ROS levels and decreases cardiac levels of Nrf2, SIRT1 and phosphorylated AMPK, leading to oxidative stress and decreased autophagy^[153]^, and activates NF-κB inflammatory signaling pathway^[154]^. In this way, D-galactose-induced aging models demonstrate LV wall thickening, cardiac fibrosis, and reduced cardiac function^[150]^.

Mutations in the *LMNA* gene cause laminopathies, a number of disorders with a wide range of phenotypes, including premature aging and cardiomyopathy^[155]^. *LMNA* gene encodes the intermediate filament proteins, lamins A and C, which are major structural components of the nuclear lamina. Homozygous *LmnaG609G/G609G* mice express lamin A, lamin C, and progerin (a truncated form of prelamin A) with the same protein expression pattern of patients with Hutchinson-Gilford progeria syndrome (HGPS), and exhibit metabolic abnormalities, cardiac electrical alterations and HFpEF, similar to the normally aged mice^[156,157]^.

Moreover, mice with mutations in the *Polg* gene, which encodes the mitochondrial DNA polymerase responsible for replication and repair of mtDNA, are also used as models of cardiovascular aging. Specifically, mice with a homozygous mutation in the exonuclease encoding domain of mitochondrial DNA polymerase gamma (*Polg*) (*Polgm/m*) develop an age-dependent accumulation of mtDNA mutations and display accelerated aging starting at 7–9 months of age. Also, they exhibit age-dependent cardiomyopathy, which is more severe than the usual phenotype of cardiac aging^[39,158,159]^, and specifically demonstrate cardiac hypertrophy, dilatation, fibrosis and dysfunction by 13–14 months of age^[153]^. Transgenic mice for a cardiac-specific mutant of *Polg* (*termed Y955C*) develop LV hypertrophy at 94 days of age^[160]^, while mice with a homozygous mutation in the encoding domain (*D257A*) of a proofreading deficient version of *Polg* develop biventricular hypertrophy at 10–12 months of age^[161]^.

## PRECLINICAL AND CLINICAL INTERVENTIONS AGAINST CARDIOVASCULAR AGING

**Figure 2.**
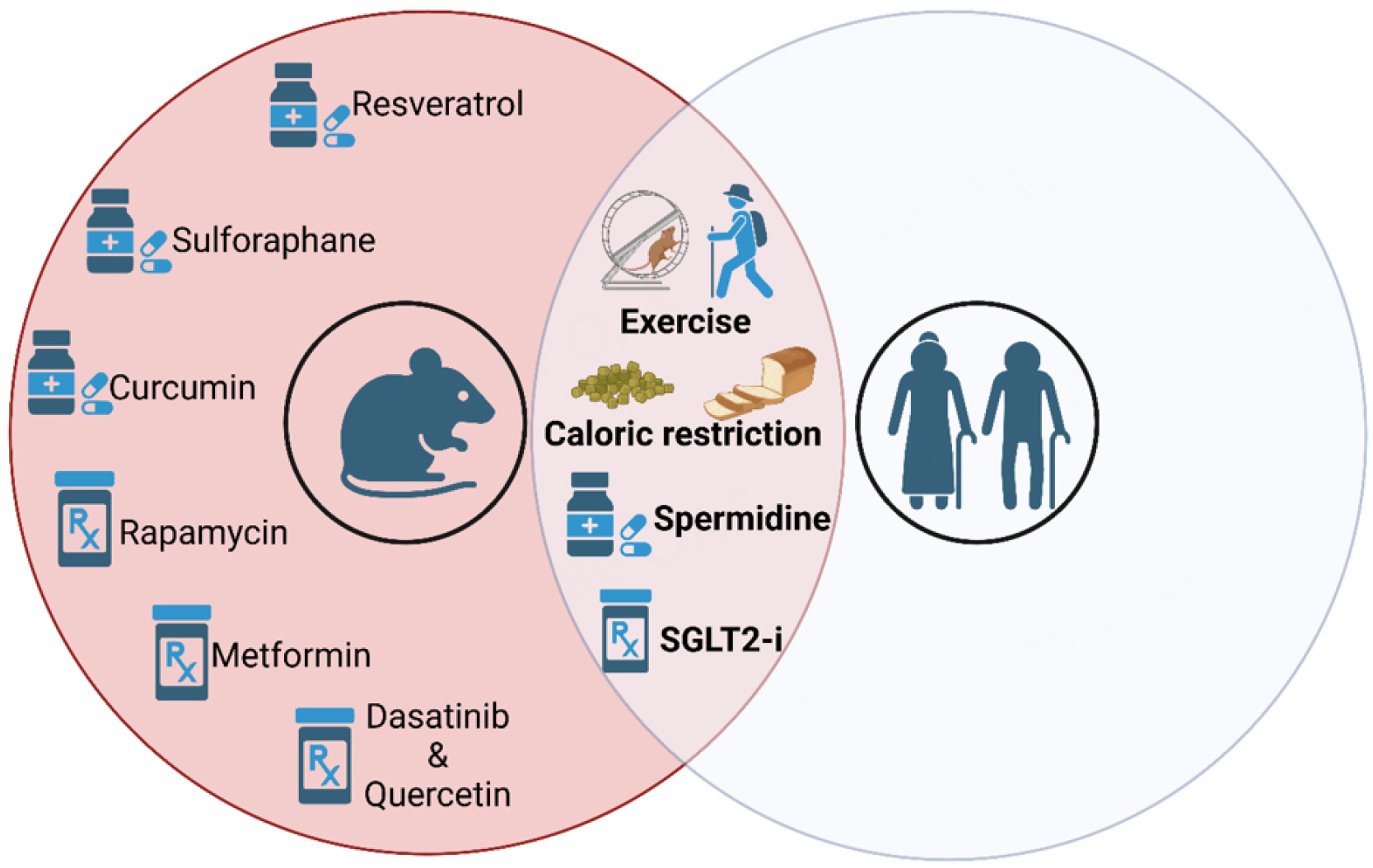
Interventions in preclinical studies that have translated into improvements in cardiac and/or vascular aging in humans (overlapping area) include exercise, caloric restriction, caloric restriction mimetics, such as spermidine, and SGLT2-inhibitors; image created on biorender.com.

Efforts aiming to tackle aging-related features of cardiomyopathy have included environmental, dietary and pharmacological interventions. Maintaining a healthy lifestyle is strongly associated with a lower risk of developing age-associated chronic diseases, including CVD. However, pharmacological agents may be needed for the prevention or treatment of CVD, especially in the elderly. Some of the interventions that showed beneficial effects in preclinical studies have been or are presently tested in humans [Figure 2].

### Exercise

Physical activity promotes healthy aging and prevents CVD^[162–165]^. Progressive and vigorous exercise for 1 year in previously sedentary people aged 65 and over induces physiological LV remodeling, increases stroke volume and total aortic compliance, and decreases arterial elastance^[166]^, while studies in aged mice correlated the benefits of exercise with increased exercise capacity, improved diastolic function, physiological cardiac hypertrophy and increased cardiomyogenesis^[167–169]^. Even in mice expressing a proofreading-deficient version of *Polg*, endurance exercise for 5 months increases mitochondrial biogenesis and mitochondrial oxidative capacity and alleviates age-associated cardiomyopathy^[170]^. Another study demonstrated that both aerobic and resistance exercise for 8 weeks improves cardiac angiogenesis and aerobic capacity in aged rats, with aerobic exercise having more profound beneficial effects^[171]^. Additionally, aerobic exercise of moderate intensity for 12 weeks restores hydrogen sulfide levels, which is an endogenous gasotransmitter that has been linked with cardioprotective properties^[172–174]^, decreases oxidative stress and lowers cardiac expression of markers of fibrosis and inflammation in aged rats^[175]^.

### Caloric restriction

A dietary approach associated with longevity is caloric restriction (CR), which is based on decreased intake of calories without malnutrition^[176]^. CR attenuates age-associated cardiac diastolic dysfunction and cardiac remodeling, as well as myocardial mitochondrial damage and lipid accumulation^[177,178]^. Enhancement of autophagy is one of the possible mechanisms via which CR protects against cardiac aging, since it activates the AMPK-forkhead box O (FOXO)-autophagy pathway and suppresses the mTOR pathway^[177,178]^. Additionally, CR partially reduces age-associated cardiac insulin resistance through the activation of the PI3K/Akt pathway in rats^[179]^. In arteries of old mice, CR can also prevent large elastic artery stiffness and endothelial dysfunction by inhibiting NADPH oxidase and increasing the activity of SOD and catalase^[180]^, which combinedly attenuate oxidative stress.

Thus, exercise decreases oxidative stress, while CR enhances autophagy, resulting in physiological LV remodeling and improved cardiac function, leading to the conclusion that a healthy lifestyle promotes healthy cardiovascular aging.

### Caloric restriction mimetics

Caloric Restriction Mimetics (CRMs) produce similar beneficial effects^[181,182]^. Resveratrol, a polyphenol found in grapes and red wine, prevents age-associated cardiac dysfunction and reverts age-related increase of inflammatory, oxidative and apoptotic markers, including TNF-α, NFκB and nitric oxide synthase, in hearts of rodents^[183,184]^. Resveratrol also improves doxorubicin-induced cardiotoxicity via restoration of SIRT1 activity in the hearts of aged SAMP8 mice^[185]^, while it ameliorates cardiac remodeling in mice with HFpEF due to inhibition of the TGF-β/Smad3 signaling pathway^[186]^.

Curcumin, a phytochemical derivative of turmeric, ameliorates vascular oxidative stress, increases nitric oxide bioavailability and alleviates age-related arterial dysfunction in mice^[187]^, as well as in healthy middle-aged and older humans^[188]^. Curcumin also induces autophagy by increasing the expression of SIRT1 and AMPK phosphorylation and decreasing mTOR phosphorylation in a dose-dependent manner^[153]^.

Polyamine spermidine is another inducer of autophagy that reverses age-associated cardiac hypertrophy, fibrosis, diastolic dysfunction and arterial dysfunction in mice^[189–191]^. Spermidine inhibits ROS accumulation and improves mitochondrial function through activation of the SIRT1/peroxisome proliferator-activated receptor-γ coactivator-1α (PGC-1α) signaling pathway in the myocardium of aged rats, leading to attenuation of cardiac senescence^[192]^. A prospective, population-based cohort study associated dietary intake of spermidine with reduced risk of fatal heart failure, acute coronary artery disease, and death due to vascular disease^[190]^.

Additionally, sulforaphane, an antioxidant found in cruciferous vegetables, upregulates NRF2 activity, improves mitochondrial function, enhances autophagy, has beneficial effects on insulin resistance, and reverses cardiac dysfunction in aged mice^[193]^.

### Dasatinib and quercetin

The combination of dasatinib and quercetin (D+Q) induces senolysis, which is the process of selective removal of senescent cells, and is considered to have beneficial effects in age-related pathologies^[194]^. D+Q administration for 1 month improves cardiac remodeling and diastolic function by removing senescent cardiac non-myocyte and myocyte cells and cardiac stem/progenitor cells in aged female mice after myocardial infarction^[195]^, whereas longer (3 months) treatment with D+Q ameliorates vasomotor function by increasing NO bioavailability and reduces aortic calcification in aged mice^[196]^. Although systolic dysfunction is not a common feature of cardiac aging in mice, another study found that EF is also improved as soon as 5 days after a single dose of D+Q in aged mice^[197]^.

### mTOR inhibitors

Rapamycin, an FDA-approved mTOR inhibitor, improves both age-related cardiac systolic and diastolic function in mice^[198]^ and dogs^[199]^. This improvement in diastolic function, along with a reduction in cardiac hypertrophy and passive stiffness, is observed in aged mice, even 8 weeks after discontinuation of an 8-week treatment^[200]^. Improvement in cardiac and skeletal muscle function by rapamycin is also seen in *Lmna*^−/−^ mice^[201]^. Moreover, rapamycin ameliorates arterial dysfunction via suppression of oxidative stress, activation of arterial AMPK, and increased PTEN expression in old mice^[64]^.

### Metformin

Metformin is one of the most widely prescribed anti-hyperglycemia drugs and is expected to be tested for slowing the incidence of age-associated multi-morbidity in the TAME (Targeting Aging with Metformin) clinical trial^[202,203]^. Metformin has been shown to improve age-related metabolic and nonmetabolic derangements in skeletal muscle and subcutaneous adipose tissues of older glucose-intolerant adults^[204]^. Preclinical studies showed that treatment of aged male mice with metformin for 28 days enhances autophagic flux in the aging vasculature, reduces SASP in senescent VSMCs, and ameliorates age-associated structural and functional changes in arteries^[205]^. Moreover, metformin-based activation of cardiac AMPK for two weeks decreases the activity of mTOR and reduces ER stress, while it decreases cardiac injury during ischemia and reperfusion in aged male hearts^[206]^. However, another study with a lower dosage of metformin showed that metformin treatment till death in aged female mice does not improve cardiac function and shortens the median lifespan^[207]^.

### Sodium-glucose cotransporter 2 inhibitors

Sodium-glucose cotransporter 2 (SGLT2) inhibitors (SGLT2-I; gliflozins) lower blood glucose by blocking glucose reabsorption in the proximal convoluted tubule of the kidney and increasing glycosuria. Except for these effects, SGLT2-I have been associated with suppression of cellular senescence and inflammaging^[208]^. At the same time, considering their benefits regarding cardiovascular events, cardiovascular and all-cause mortality in aged adults^[209]^, SGLT2-I may also have anti-aging properties.

Empagliflozin attenuates age-related endothelial dysfunction and arterial stiffening, and reduces vascular oxidative stress in aged mice^[210]^, while in mice with streptozotocin (STZ)-induced diabetes, it improves cardiac function by decreasing cardiac fibrosis and senescence^[211]^. Additionally, empagliflozin improves cardiac mitochondrial function, reduces cardiac fibrosis and increases the survival rate in mice with heart and skeletal muscle-specific manganese superoxide dismutase (MnSOD)-deficiency (MnSOD-cKO mice under the control of the muscle creatine kinase promoter)^[212]^, which is a mitochondrial antioxidant enzyme.

Mice older than 18 months with HFpEF phenotype due to exposure to high fat diet (HFD) and Ang II demonstrate improved global longitudinal strain (GLS) and decreased cardiac fibrosis after dapagliflozin treatment^[213]^. However, the same study showed that liraglutide, a glucagon-like peptide-1 receptor agonist, leads to more significant improvement in cardiac function, and it reduces cardiac hypertrophy and fibrosis to a greater extent than dapagliflozin^[213]^. Dapagliflozin also improves action potential repolarization and restores Ca^2+^ homeostasis in aged cardiomyocytes^[214]^.

Recently, the EMPEROR-Preserved and DELIVER clinical trials showed the beneficial properties of empagliflozin and dapagliflozin in patients with HFpEF, respectively, in terms of lower combined risk of cardiovascular death or hospitalization for heart failure^[215,216]^.

## EPILOGUE-CONCLUSIONS

Cardiovascular aging is characterized by structural and functional changes in the cardiovascular system that include cardiac hypertrophy, diastolic dysfunction, myocardial fibrosis, arterial stiffness, endothelial dysfunction, and valvular calcification. Oxidative stress, mitochondrial dysfunction, impaired autophagy, inflammaging, fibrosis, and telomere dysfunction are the main cellular processes that cause these cardiovascular changes. Accordingly, proteins that are involved in ROS formation and accumulation are increased. The consequences of oxidative stress are further exacerbated due to impaired autophagy/mitophagy that would facilitate the removal of damaged organelles, such as mitochondria.

Besides the molecular pathways that regulate these processes, the advances in the field of cardiovascular aging also involve possible pharmacological and lifestyle interventions. Exercise and CR reduce oxidative stress and enhance autophagy in the hearts and vessels of preclinical models, while CRM, such as resveratrol, curcumin, spermidine, and sulforaphane, produce similar beneficial effects, resulting in improved cardiac diastolic and arterial function. Furthermore, antidiabetic drugs, such as metformin and SGLT2-I, ameliorate age-associated cardiac and arterial dysfunction by decreasing oxidative stress, inflammation and fibrosis. TAME clinical trial testing if metformin will alleviate age-associated multimorbidity is expected to launch, while clinical trials with SGLT2-I proved their benefit in patients with HFpEF.

As the understanding of the biology of aging becomes deeper, translational research is expected to indicate more novel therapies that decelerate the rate of cardiovascular aging.

## Figures and Tables

**Table 1.** Animal models of cardiovascular aging

Model	Relevance to aging	Cardiovascular phenotype	Refs.

**1. Natural aging**			
Aged C57BL/6J mice		Cardiac hypertrophy and dysfunction, enhanced fibrosis, aortic stiffness	[217,218]
Aged Fischer 344 rats		LV hypertrophy, diastolic dysfunction, abdominal aorta with decreased sodium nitroprusside-mediated relaxation	[219,220]
Aged Fischer 344/Brown Norway F1 rats		LV dilatation, mild hypertrophy, fibrosis, LV diastolic and systolic dysfunction	[221]
Aged Rhesus macaque (Macaca mulatta)		Myocardial fibrosis, cardiac hypertrophy, aortic valve calcification	[146]
**2. Premature aging**			
Senescence-accelerated prone (SAMP) mice	Selective inbreeding of AKR/J mice with inherited senescence	Cardiac diastolic dysfunction, cardiac fibrosis, vascular dysfunction	[148,222]
Mus musculus castaneus (CAST)	Mice with short telomeres from birth	Cardiac dysfunction, hypertrophy, fibrosis and senescence	[149]
**3. Chemically induced aging**			
D-galactose-induced aging in mice and rats	D-galactose treatment increases the levels of senescence-associated β-galactosidase (SA-β-gal)	Increase in heart weight, cardiac hypertrophy, cardiac fibrosis and LV dysfunction	[150]
**4. Progeria models**			
*LmnaG609G/G609G mice*	*LMNA* point mutation (*G609G*) that activates a cryptic donor splice site and produces progerin, a truncated form of prelamin A	Hutchinson-Gilford progeria syndrome (HGPS), cardiac fibrosis, electrocardiographic alterations, cardiac diastolic dysfunction, HFpEF	[157]
*LmnaN195K/N195K* mice	*LMNA* missense mutation (*N195K*) in lamins A and C	Dilated cardiomyopathy with conduction system disease	[223]
*LmnaH222P/H222P* mice	*LMNA* missense mutation (*H222P*) identified in a family with autosomal dominant Emery-Dreifuss muscular dystrophy (EDMD)	EDMD, DCM	[224]
Hypomorphic *BubR1 mutant*(*BubR1H/H*) mice	Reduced expression of spindle assembly checkpoint kinase BubR1, leading to chromosome number instability	Cardiac arrhythmias, arterial wall stiffening	[225,226]
**5. Mitochondrial mutations**			
Cardiac-specific termed *Y955C Polg* mutant mice	*Polg* point mutation (*Y955C*) leading to mtDNA depletion	LV hypertrophy, increased ventricular volume	[160,227]
*D257A Polg* mutant mice	*Polg* mutation (*D257A*) in the N-terminal "proofreading” exonuclease domain, resulting in a protein without polymerase proofreading function in mitochondria	Biventricular hypertrophy, fibrosis, heart failure, arterial stiffening	[161,228]
Tafazzin-deficient mice	Mice lacking tafazzin, a mitochondrial transacylase essential for cardiolipin remodeling	Barth syndrome, LV dilation, reduced ejection fraction	[229]
heart/muscle-specific Mn-SOD-deficient mice (*H/M-Sod2^−/−^*)	Mice with a specific in the heart and skeletal muscle loss of manganese superoxide dismutase (Mn-SOD) expression, a principal scavenger enzyme in mitochondrial matrix	Dilated cardiomyopathy, reduced cardiac contractility	[230]
**6. Other global gene mutations**			
Klotho-deficient (*KL^−/−^*) mice	Knock-out of Klotho	Impaired cardiac function, increased heart size, increased LV myocardial mass	[57]
G5 telomerase-deficient (telomerase RNA component, *TERC^−/−^*) mice	Telomerase-deficient mice after G3 generation have short telomeres, aneuploidy, and end-to-end chromosome fusions	Pathological cardiac remodeling, severe ventricular dysfunction	[231,232]
Pim triple knock-out mice (*Pim1, Pim2 ^−/−^ and Pim3 ^−/−^*)	Mice lacking Pim kinases, which are highly conserved serine/threonine kinases, have altered mitochondrial morphology	Cardiac hypertrophy, heart failure, cardiac fibrosis	[233]
Interleukin-10 knockout *IL-10*(*tm/tm*) mice	Mice lacking IL-10, which is an anti-inflammatory cytokine	Stiffer vessels, reduced vascular relaxation, asymmetric hypertrophy, systolic and diastolic dysfunction	[234]
DNA-helicase-regulatory protein (*WRN*) *WRN-K577M* mutant mice	Amino acid substitution of WRN at position 577 eliminates the ATPase and helicase activity	Werner syndrome, diastolic LV dysfunction, cardiac fibrosis and hypertrophy	[235]

homozygous mutation in the encoding domain *(D257A)* of a proofreading deficient version of *Polg* develop biventricular hypertrophy at 10–12 months of age^[161]^.

## Data Availability

Not applicable.
